# Differences in Mechanical Parameters of Keyboard Switches Modulate Motor Preparation: A Wearable EEG Study

**DOI:** 10.3389/fnrgo.2021.644449

**Published:** 2021-08-18

**Authors:** Hiroki Watanabe, Kae Nakajima, Shunsuke Takagi, Ryo Mizuyama, Mayumi Saito, Koichi Furusawa, Kunio Nakatani, Yusuke Yokota, Hirotaka Kataoka, Hiroshi Nakajima, Yasushi Naruse

**Affiliations:** ^1^Center for Information and Neural Networks, National Institute of Information and Communications Technology, and Osaka University, Kobe, Japan; ^2^OMRON Corporation, Kyoto, Japan

**Keywords:** contingent negative variation, motor preparation, electroencephalography, event-related potentials, neuromarketing

## Abstract

The mechanical parameters of keyboard switches affect the psychological sense of pressing. The effects of different mechanical parameters on psychological sense have been quantified using questionnaires, but these subjective evaluations are unable to fully clarify the modulation of information processing in the brain due to these differences. This study aimed to elucidate the ability of electroencephalography (EEG) measurements to detect the modulation of subconscious information processing according to mechanical parameter values. To this end, we prepared five mechanical switches with linearly increasing values of pretravel (PT: the distance from the free position until the operating position). We hypothesized that the differences in PTs would subconsciously affect the motor preparation prior to pressing switches because switches with PTs that deviated from those commonly used were predicted to increase the users' attention level when pressing. Differences in motor preparation were quantified using the mean amplitudes of the late contingent negative variation (CNV). We recorded EEGs of 25 gamers during a reaction task for fast switch pressing after a response cue preceded by a pre-cue for response preparation; we also measured the reaction time feedback on each switch pressing trial. Participants performed five sessions (60 trials per session) in total. For the analysis, trials were divided into first (session 1, 2, and 3) and second half sessions (session 4 and 5). In the latter session, CNV amplitudes were significantly higher for the switch with the highest PT than for that with a medium PT, which is closest to that commonly used in commercial mechanical switches. On the other hand, the questionnaire did not detect any significant differences between PTs in their subjective rankings of the psychological effects of switch pressing. These results suggest that differences in PTs modulate motor preparation to press switches, and that EEG measurements may provide a novel objective evaluation of the mechanical parameters of keyboard switches.

## 1. Introduction

The quantification of the psychological feelings of products plays a crucial role in evaluating the product design process to ensure the development of consumer-oriented products (Nagamachi, 1995). To date, questionnaire surveys such as the rating scale method (raters select an applicable level on a 5- or 7-scale for each question item), paired comparisons (Thurstone, 1927; Scheffé, 1952), or semantic differential method (Osgood et al., 1957) have been utilized in evaluation of the design for quantification purposes. Although these subjective evaluations have the advantage of gathering a large amount of data in a relatively short period, raters' biases may hinder reliable measurements. For example, social desirability bias tends to lead raters to choose the answers that are favorably recognized by others (Furnham, 1986; King and Bruner, 2000). Further, subjective evaluation generally fails to quantify raters' exact feelings on the products, as emotional processes in the brain may be induced subconsciously (Kiss and Eimer, 2008; Pantazatos et al., 2012), and subconscious processing affects the consumption behavior of consumers (Winkielman et al., 2005).

A potential approach for more accurate and reliable evaluation of products is the incorporation of brain activity measurements into the product design process. Neuromarketing or consumer neuroscience has received substantial attention as a field focusing on the objective quantification of consumers' feelings, preferences, and cognitive processing of products or product advertising based on brain activity for product marketing (Ariely and Berns, 2010; Morin, 2011; Spence, 2019). Extensive research has demonstrated that machine-learning technology enables the estimation of emotional states using brain responses (Wang et al., 2014; Aldayel et al., 2020; Liu et al., 2020). In addition, measurements of blood-oxygen-level dependent (BOLD) signals using functional magnetic resonance imaging (fMRI) or electroencephalogram (EEG) enabled marketers to clarify the brain activity patterns underscoring consumers' willingness to pay for products (Plassmann et al., 2007; Ramsøy et al., 2018). Furthermore, for objective evaluation of products, Guo et al. (2020) indicated that the measurement of N400, an event-related potential (ERP) associated with semantic processing, could be used to identify adjectives that well describe products. For neuromarketing research, there are several ways to measure brain activity. MRI scanners are not suitable for real-world environments research due to their large size, but EEG is compact and has relatively low running costs, making it suitable for real-world neuromarketing research (Bazzani et al., 2020) such as an evaluation of a beverage machine in an office environment (Sargent et al., 2020).

Given the relevance of brain state information for the objective evaluation of products in neuromarketing, its measurement may be useful for evaluating the mechanical parameters of industrial products. In particular, we focused on incorporating brain states into the evaluation of the mechanical parameters of mechanical keyboard switches. Mechanical switches include various mechanical parameters such as pre-travel (PT), which corresponds to the distance traveled by the switch when moving from a position where external force is not applied (i.e., the free position) to another when it is pressed (i.e., the operating position); the operating force, which is the force required to move the switch from the free position to the operating position; and total travel, the travel distance from the free position to the switch limit position (Supplementary Figure 1). Recently, various types of mechanical switches with different mechanical parameters have been attracting attention in the pursuit of performance in e-sports. Among those parameters, PT is particularly difficult to evaluate because PT differences do not change any physical feeling while pressing switches (i.e., the difference is just a distance), and thus, users cannot explicitly notice PT differences just by switch pressing. Thus, the method to evaluate the optimal PT value in switch design is to receive feedback on the reaction times (RTs) when pressing switches with different PT values. However, there is a possibility that inappropriate PT values require users to increase their attention to switch operations. For a long PT, the RT would be longer than necessary, and the switch considered difficult to operate for quick responses. Given that RTs are correlated with sustained attention in reaction tasks (Buck, 1966), a longer PT might modulate the allocation of attentional resources to switch operation for quicker responses than switches with normal PT values. Also, switches with extremely short PTs tend to produce unexpectedly fast RTs, and such unpredictability may increase the allocation of attention to switch operations. Based on the above, we predicted that switches with largely different PT values from commonly used ones would require more attentional resources during motor preparation due to unpredictable responses. If so, adding the estimation of the attention allocated to switch operation to switch evaluation criteria would be beneficial to prevent the increase in attentional resources during the gameplay. Therefore, the present study aimed to investigate whether pressing switches with deviated PT values induces an increase in attentional resources in switch operations, and whether such an increase can be detected using EEG measurements.

If, as expected, differing PT values from commonly used ones increase the allocation of attentional resources during switch operation, differences in PT values would be expected to be reflected in the amplitude of contingent negative variation (CNV) during motor preparation before switch pressing. This ERP component is characterized by a sustained negative deflection during motor preparation before a response cue (the target cue) preceded by a pre-cue for response preparation (the pre-cue) (Walter et al., 1964). The CNV consists of early and late components, with the late one reflecting the participants' motor preparation to a target cue (Gaillard, 1977; Leuthold et al., 2004). Previous research has demonstrated that an increase in attention during motor preparation enhances late CNV amplitude (McCallum and Walter, 1968; Tecce, 1972) and CNV has been used to investigate attentional mechanisms in motor response tasks (Liebrand et al., 2017). If the difference in PT affects the user's attention to the switch operation, then the difference in PT values would modulate the late CNV amplitude. However, it remains unclear whether this measure of brain activity allows the assessment of an increase of the attentional resources allocated to switch operation induced by PT differences.

To assess the validity of including the attentional resources allocated to switch operation into the evaluation criteria for mechanical switches, we investigated whether different PTs increase the attentional resources during motor preparation for switch pressing and thus modulate late CNV amplitude. To this end, we prepared five experimental switches with different PTs linearly increasing from Switch 1 (shortest PT) to Switch 5 (longest PT), and Switch 3 corresponding to the ordinary PT range in commercially available switches, while maintaining other mechanical parameters constant. If differences in PT modulate the attentional resources allocated to switch operation and the late CNV is a valid indicator of this change, then we predicted that the mean late CNV amplitude would be enhanced when pressing a switch with a largely different PT from normal switches (i.e., Switch 1 or Switch 5; switches with shorter or longer PT; see section 2.2 for details). In order to induce the late CNV, subjects performed a reaction task in which they had to press these switches as quickly as possible, preceded by a pre-cue to indicate which switch to press among all five switches. Due to the purpose of the current study, data were collected from subjects with experience using gaming keyboards. Since it is not possible to notice differences in PT values just by the sensation of switch pressing, auditory and visual feedback of RTs was provided so that participants implicitly noticed the gap between predicted and actual RTs with session progression. To consider such implicit learning throughout sessions, we divided the trials into a total of five sessions with first half (session 1, 2, and 3) and second half sessions (session 4 and 5) for data analysis.

We also examined whether differences in PT values are reflected in other EEG responses via implicit learning. First, we expected that the parietal pre-cue-locked P3 would also be modulated according to the switch type since a direct relationship between PT values and RT speed could result in a reward prediction that shorter PT switches tend to score better on the task. Given that task-relevant, reward-predictive cues increase P3 amplitude (Krebs et al., 2013; Schevernels et al., 2014, 2016; Carsten et al., 2021), it is likely that differences in subjective reward prediction across PT values modulate the amplitude. We also analyzed feedback-related P3, which is distributed in the parietal region and reflects outcome evaluation and reward processing (Yeung and Sanfey, 2004; Hajcak et al., 2005, 2007; Wu and Zhou, 2009), as this component is known to be enhanced when subjects put more effort in difficult tasks (Ma et al., 2014; Schevernels et al., 2016). If subjects change their effort due to PT differences, the amplitude of feedback-related P3 might also be modulated. Second, the gap between the actual and predicted RTs when pressing a switch with deviating PTs might direct participants' attention to the feedback and modulate feedback-related ERPs. Since top-down attention enhances evoked auditory ERPs such as N1 (Hillyard et al., 1973; Näätänen and Picton, 1987), we analyzed the N1 response evoked by the feedback sound. Finally, we measured frontal theta power before switch pressing as an EEG index to quantify the mental workload (Sammer et al., 2007; So et al., 2017). Since the change in the power has applied as an index of mental workload in real-world neuroergonomics research (e.g., Aricó et al., 2016), we also measured this spectral power during motor preparation in order to contrast our proposed CNV index with the index often used in the research field. After the EEG measurements, to determine whether a questionnaire survey could also detect differences in PT values, subjects were asked to respond to a subjective evaluation of each switch.

## 2. Methods

### 2.1. Participants

To obtain data from participants with sufficient computer gaming experience using a gaming keyboard, we recruited participants who met both of the following conditions: an individual who (1) has purchased a gaming keyboard before, and (2) has played any of First Person Shooter (FPS), Third Person Shooter, Multiplayer Online Battle Arena, Multiplayer Online Role-Playing Game, and Massively Multiplayer Online Role-Playing Game over 50 h.

In total, 25 right-handed males participated in the current study (age range: 20–35, mean age = 25.8, SD = 5.0). Participants had normal or corrected-to-normal vision and reported no medical history of mental disorders, attention deficit disorder, or neurological disorders. The current study was approved by the Ethical Committee for Human and Animal Research of the National Institute of Information and Communications Technology. Participants agreed to participate in the current study and provided written informed consent before the experiment.

The total number of participants was determined based on a pilot study (*N* = 9) to analyze the differences in the mean amplitude of late CNV between switches with short, medium, and long PT values. The results of the pilot study showed that the effect size (Cohen's *d*) of the difference in late CNV between switches with short and medium PT values and between switches with long and medium PT values was 0.68 and 0.93, respectively. Power analysis at an alpha level of 0.05, statistical power of 0.8, and an effect size of 0.68 using G*Power 3.1 (Faul et al., 2007) showed that 19 participants were needed for the paired *t*-test. In addition, a medium effect size of *d*_*z*_ 0.58 was detectable for the paired *t*-test with the current number of participants.

### 2.2. Apparatus

**Figure 1:**
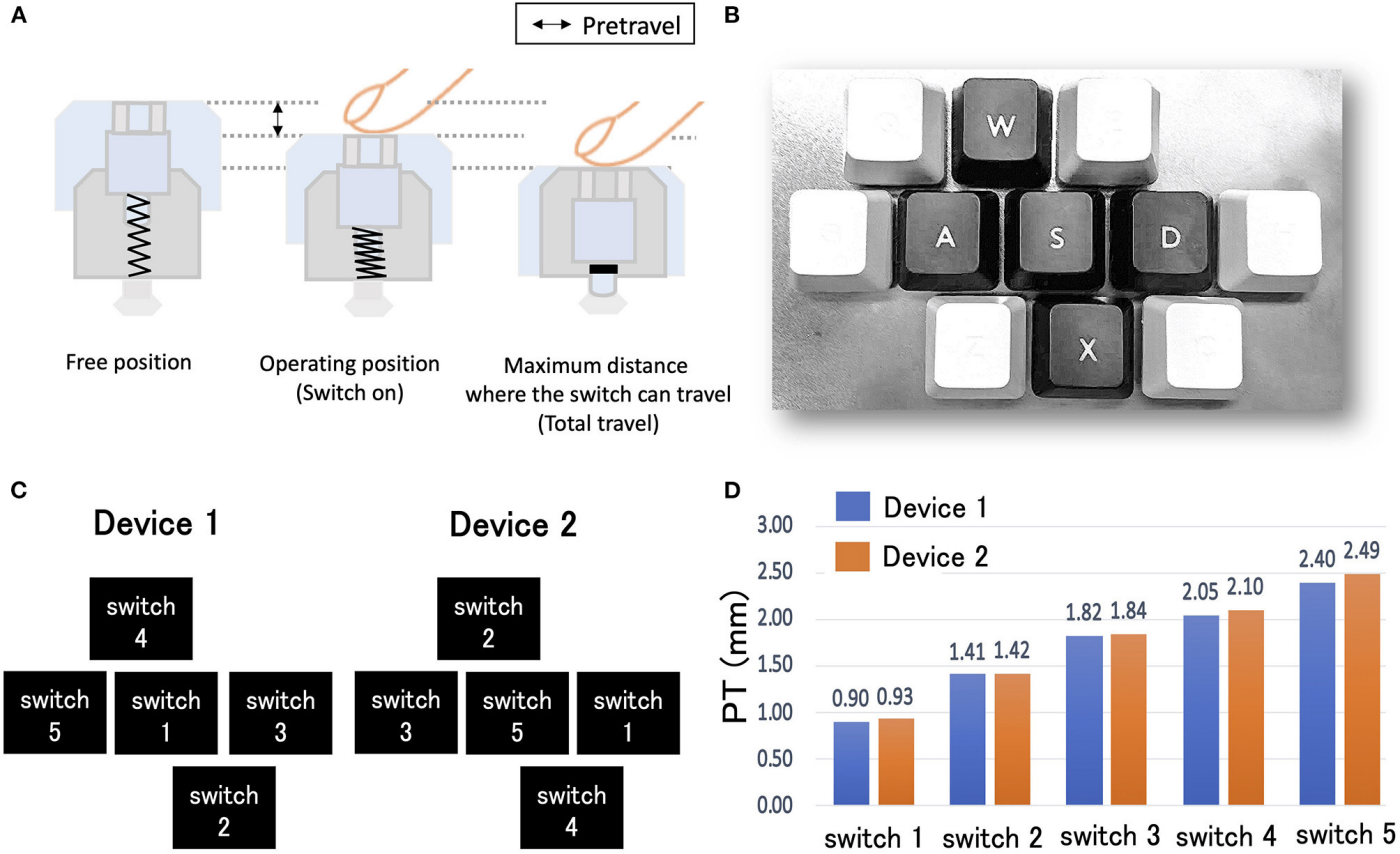
**(A)** Schematic explanation of pre-travel (PT) value of a mechanical keyboard switch. PT represents the distance from a free position to an operating position of a switch. **(B)** An image of the experimental device. The five experimental switches were set on the metallic board. The arrangement of the experimental switches follows the positions of A, S, D, W, and X of a commercially available keyboard. **(C)** To avoid the potential confounding effect of switch position on ERP amplitudes, two experimental devices were prepared with the experimental switches set at different positions. In both devices, the experimental switches 1, 3, and 5 were set in the horizontal positions (i.e., A, S, and D positions); and switches 2 and 4 were set in the upper or lower positions (i.e., W and X positions). **(D)** PTs of the five experimental switches in devices 1 and 2. The PTs of both devices increased linearly from Switch 1 to 5.

To investigate the modulation of EEG responses according to mechanical parameters of keyboard switches, we prepared five experimental switches with different PTs. Figure 1A depicts a schematic explanation of the PT of the mechanical switches. The PTs of the experimental switches increased linearly from Switch 1 with the shortest PT to Switch 5 with the longest PT. The experimental switches were mounted on a metallic board to follow the A, S, D, W, and X key positions of a commercially available keyboard (Figure 1B). These keys were chosen because they are frequently used to move characters in FPS games. To prevent a switch position from affecting ERP amplitudes, we prepared two switch devices which set the experimental switches in different positions. In both devices, experimental switches 1, 3, and 5 were set in the horizontal positions (i.e., A, S, and D positions); and switches 2 and 4 were set in the upper or lower positions (i.e., W and X positions; Figure 1C). Half of the participants used device 1, and the remaining participants used device 2. Figure 1D depicts the PTs of the experimental switches in both devices. The PT of Switch 3 was considered the closest to the commonly used value in mechanical switches based on an investigation of the PTs of 57 commercially available mechanical switches which revealed a mean value of 1.80 ± 0.35.

EEGs were measured using a wireless wearable system (PolymateMini AP108; Miyuki Giken Co., Ltd., Tokyo, Japan) with dry electrodes (Unique Medical Co., Ltd., Tokyo, Japan). The electrodes were positioned on Fz, Cz, and Pz sites according to the International 10-20 system. The ground and reference electrodes were set on the left and right earlobe, respectively. To monitor eye-related activity, horizontal and vertical electrooculograms (EOGs) were recorded from electrodes placed on the top and side to the lateral canthus of the participants' left eyes. All signals were sampled at 500 Hz.

### 2.3. Data Collection

**Figure 2:**
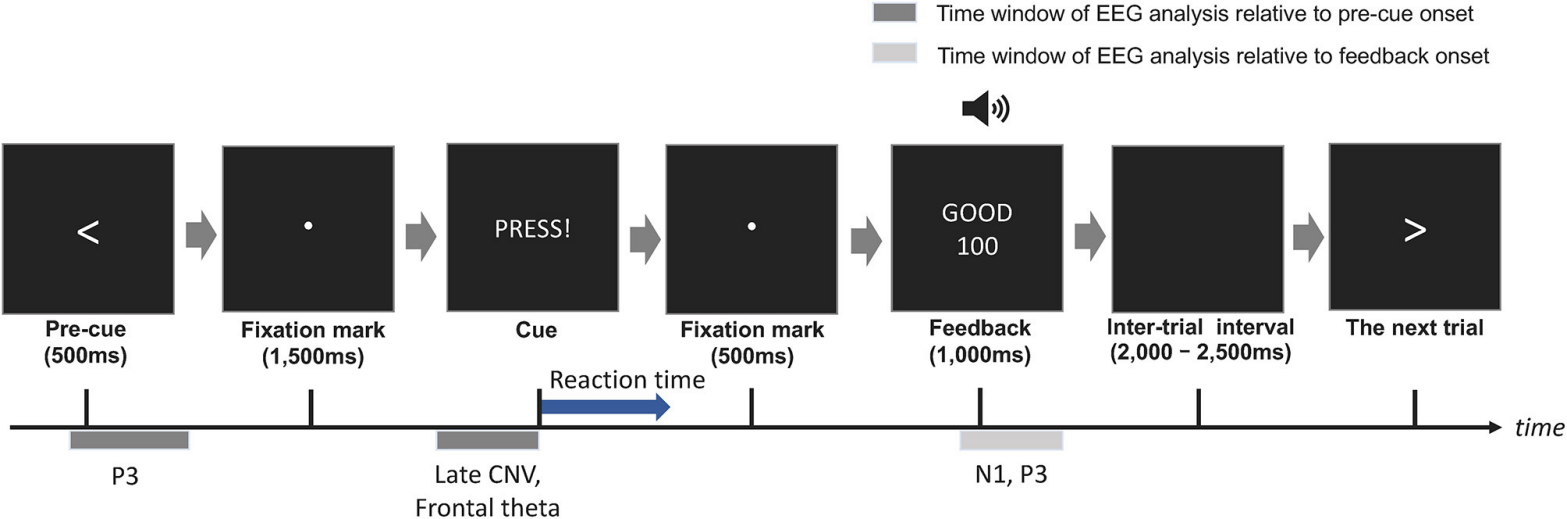
Reaction task procedure for pressing the experimental switches. Participants pressed the target switch as soon as possible after the cue (“PRESS!”) was presented at the center of the display. Auditory and visual feedbacks regarding their RTs was presented on every trial.

Participants sat on a comfortable chair in a dimly lit soundproof room and performed a reaction task to press the experimental switches. The monitor display and switch device were set in front of the participants. Because the A, S, D, W, and X keys are assigned on the left side of the keyboard, in order to reproduce the key presses during actual game play, we asked the participants to press switches with their non-dominant left hand. A trial procedure is summarized in Figure 2. Participants placed their left middle finger on the center switch (i.e., S switch) before the start of each trial. First, a pre-cue to indicate the switch to be pressed by the participants (i.e., target switch) appeared at the center of the display for 500 ms. The pre-cues >, ≡, <, ∧, and ∨ represented the right, center, left, upper, and lower switches on the experimental device (i.e., D, S, A, W, and X switches; cf. Figure 1B), respectively. Participants set their left middle finger on the target switch to prepare for a response. To indicate a fixation point, the pre-cue was replaced with a white dot for 1,500 ms. Participants pressed the target switch as quickly as possible after the presentation of the cue string “PRESS!.” The cue was changed back to the fixation mark immediately after the participants' response or automatically 500 ms after the onset of the cue. Visual and auditory feedback on the participant's responses was presented 500 ms after the participant's response and the visual feedback lasted for 1,000 ms. The inter-stimulus interval from the feedback end to the beginning of the next trial was randomly set from 2,000 to 2,500 ms. The interval was chosen to avoid contamination of feedback-related components with the baseline time region for ERP calculation. Participants were instructed to avoid blinking as much as possible during the period from the onset of a pre-cue to the onset of feedback to avoid contaminating EEG data with eye-related activity.

During the feedback section in a trial, an earned point and response category were presented on the display. These parameters were calculated based on participants' RT. The response categories were *False start* (RT <150 ms), *Good* (150 ms ≤ RT <330 ms), *Late* (330 ms ≤ RT <700 ms), and *Over* (700 ms ≤ RT). The time range of *False start* was chosen given that simple RT generally falls within 200–300 ms (e.g., Eckner et al., 2010). The trials in which participants pressed a non-target switch were categorized as *Error*. The earned point was calculated using Equation (1):


(1) $$points=\{\begin{array}{l} -500\\ \;(response=False\;start\;or\;response=Over)\\ \frac{300\times (RT_{upper}-RT)}{RT_{upper}-RT_{lower}}\\ 12pt(response=Good)\\ -100\\ \;(response=Late\;or\;response=Error) \end{array}$$

where *RT*_*lower*_ and *RT*_*upper*_ are the lower and upper limits of RTs in *Good* trials (i.e., 150 and 330 ms), respectively. In the case of *Good* trials, the equation transformed participants' RT in a range from 0 to 300 points. In the feedback section, feedback on whether the RT was categorized as *Good* or not was provided aurally, and the category of participants' responses and earned points were presented on the display. After completion of a trial session, the total points across switches and per switch were displayed on the display. Participants performed 12 trials per switch in a session (i.e., 60 trials/session). The order of trials was randomized per session and participant. Participants performed one practice session and five main sessions. The EEG data collection procedure, including recording preparation, lasted ~60 min.

After the reaction task, a questionnaire survey was administered to investigate the subjective feelings of pressing the switches. Participants ranked the switches according to each question item on a 5-point scale while referring to the mean earned points of each switch across sessions (1: the best; 5: the worst). Participants were allowed to press the experimental switches anytime when answering the questions to re-confirm the feelings of the switches. A tied rank was assigned for cases in which participants judged that there was no difference in subjective feelings between switches. The question items are summarized in Table 1.

**Table 1:** The question items in the questionnaire survey.

**Question item**	**Explanation**
1. Pleasantness of the pressing sound	The sound when pressing the switch feels pleasant.
2. Pitch of the pressing sound	The sound when pressing the switch has a high pitch.
3. Loudness of the pressing sound	The sound when pressing the switch feels loud.
4. Response speed	The response of the switch feels quick.
5. Niceness of responses	The response of the switch feels nice.
6. Click feeling	The switch clicks when pressing.
7. Lightness	The switch feels light when pressing.
8. Operability	The operability of the switch feels good.
9. Suitability for gaming	The switch feels suitable for gaming purposes.
10. Comfortableness	The switch feels comfortable when pressing.
11. Reliability	The switch feels reliable to use.
12. Satisfaction	The switch is satisfying to use.
13. Attractiveness	The switch feels attractive.
14. Liking	The switch is to my liking.

### 2.4. EEG Analysis

We used the EEGLAB toolbox (Delorme and Makeig, 2004) for MATLAB (The MathWorks, Inc., U.S.A) to analyze EEG data. The data were preprocessed separately per participant and session. Raw continuous EEG and EOG data were off-line filtered using a 16,500th finite impulse response (FIR) high-pass filter at 0.1 Hz and a 330th low-pass FIR filter at 20 Hz. The artifacts of the raw data were suppressed using an Artifact Subspace Reconstruction method (Mullen et al., 2015) using an open-source plug-in function *clean_rawdata* in the toolbox. To correct for eye-related activity, data were decomposed using independent component analysis. The components related to eye movements and blinks were determined by visual inspection, and data were reconstructed using the components excluding the eye-related components.

To analyze data where participants appropriately responded to switches, we used the “*Good*” trials for further analysis. To observe the effects of switch types on both EEG responses relative to pre-cue onset and feedback onset, we analyzed data separately per onset type. The data were split in epochs in a range [−500, 2,100 ms] and [−100, 600 ms] relative to the pre-cue and feedback onsets, respectively. Since differences in the mean amplitude of the baseline period across conditions influence the post-stimulus ERPs, we employed a two-way repeated measures analysis of variance (ANOVA; channel × switch) for each type of dataset (i.e., pre-cue onset and feedback onset) to determine if the mean amplitude of the pre-stimulus time window in ERPs differed across conditions. Since Mauchly's sphericity test revealed a violation of the sphericity assumption in all main effects and interactions in both datasets (*p* < 0.05), the degrees of freedom were corrected by the Greenhouse-Geisser epsilon. No significant main effects and interactions were observed in the pre-cue onset [switch: *F*_(2.03, 48.79)_ = 0.56, *p* = 0.58, channel: *F*_(1.59, 38.05)_ = 2.48, *p* = 0.11, switch × channel: *F*_(1.80, 43.29)_ = 1.03, *p* = 0.36] and feedback onset dataset [switch: *F*_(2.11, 50.65)_ = 0.32, *p* = 0.74, channel: *F*_(1.54, 36.93)_ = 0.24, *p* = 0.73, switch × channel: *F*_(1.85, 44.50)_ = 0.88, *p* = 0.42]. The epoch trials were baseline-corrected using the pre-stimulus region [−100, 0 ms]. In each dataset, epochs including signals exceeding a range of ±80 μv were rejected from further analysis. We excluded one participant's data from the pre-cue onset dataset as the number of remaining trials was <20 in all switch conditions due to a low signal-to-noise (S/N) ratio in ERP data. To confirm whether the number of rejected trials differed significantly across switches, the number of rejected trials was submitted to a one-way repeated measures ANOVA. Since Mauchly's sphericity test revealed a violation of the sphericity assumption (pre-cue onset: *W* = 0.0001, *p* < 0.01, feedback onset: *W* = 0.04, *p* < 0.01), the degrees of freedom were corrected by the Greenhouse-Geisser epsilon. No significant main effect was observed [pre-cue onset: *F*_(1.34, 30.73)_ = 0.06, *p* = 0.87, feedback onset: *F*_(1.48, 35.62)_ = 2.53, *p* = 0.11]. To consider the time-series variation of EEG modulations by the implicit learning of differences in the mechanical parameters, the EEG trial data in sessions 1, 2, and 3 were categorized as session 1 and the remaining sessions (session 4 and 5) as session 2. Single participant ERP waveforms were obtained by averaging across trials per participant, session, and switch types in each dataset.

We identified the mean amplitudes of three types of ERP components: N1 (auditory feedback onset), P3 (auditory feedback onset and pre-cue onset), and late CNV (pre-cue onset). For ERP analysis, a recently reported statistical analysis can determine the detailed time windows when significant effects are observed using a permutation test (Maris and Oostenveld, 2007). However, in the current study, and from the perspective of ergonomics research, we adopted the calculation of mean amplitudes in a predefined time window for simplicity. The time window of these ERPs was determined based on grand-averaged data where all switches and sessions were included (Luck and Gaspelin, 2017). To calculate the mean amplitude of the feedback-locked N1, we chose a time window of ±20 ms centered on the peak latency of the grand-averaged ERPs (N1: [98, 138 ms]). Since the auditory N1 response shows fronto-central distribution, we selected the Fz channel for N1 analysis. For the pre-cue-locked and feedback-locked P3, time windows of ±100 ms and ±50 ms, respectively, were selected centered on the peak latency of the grand-averaged ERPs (pre-cue-locked: [298, 498 ms], feedback-locked: [292, 392 ms]). The length of these time windows was determined by visual inspection of the grand-averaged ERPs. It seems that a range of these time windows is not incompatible with previous studies (Schevernels et al., 2016) that analyzed the pre-cue-locked and positive feedback-locked P3 in a motor response task involving the motor preparatory process between the pre-cue and the cue signal (pre-cue-locked: [400, 600 ms], feedback-locked: [300, 450 ms]). The Pz channel was used for P3 analysis because the component distributed parietally (Krebs et al., 2013; Ma et al., 2014; Schevernels et al., 2016; Carsten et al., 2021). We used a time window between [1,500, 2,000 ms] for the late CNV relative to the pre-cue onset (Schevernels et al., 2016). Considering that the late CNV reflects motor preparation (Leuthold et al., 2004) and is considered to partially overlap with the early CNV, we normalized the late CNV amplitudes to early CNV amplitudes [1,000, 1,500 ms] using Equation (2):


(2) $$\begin{array}{l} CNV_{normalized}=CNV_{late}-CNV_{early} \end{array}$$

where *CNV*_*late*_ and *CNV*_*early*_ are mean amplitudes of the late and early CNV, respectively. The Cz channel where the CNV is dominantly observed (Verleger et al., 2000) was used for the calculation.

To contrast the our proposed CNV index with one used in neuroergonomics research (Aricó et al., 2016), we also examined the modulation of frontal theta power as an index of mental workload before pressing the switch, using the same time window as the late CNV. To this end, the mean power across the theta frequency band (4–8 Hz) at Fz in the time window during motor response preparation [1,500, 2,000 ms] was obtained using the Fourier transform with Hanning window tapering. The frontal theta power was converted to a decibel scale relative to the theta power in the prestimulus region [−500, 0 ms] using Equation (3):

(3) $$\begin{array}{l} frontal\text{\_}theta=10log_{10}\left(\frac{power_{post}}{power_{pre}}\right) \end{array}$$

where *power*_*post*_ and *power*_*pre*_ are the frontal theta power in [1,500, 2,000 ms] and [−500, 0 ms] relative to the pre-cue onset, respectively.

### 2.5. Statistical Analysis

Because of the possibility that switch positions in the upper/lower (W and X) or horizontal position (A, S, or D) (cf. Figure 1B) affected participants' task responses, we performed a paired *t*-test between switch positions (upper/lower or horizontal position). As PT values linearly increased from Switch 1 (Shortest) to Switch 5 (Longest), the mean PT values of the upper/lower switches (Switch 2, 4) and horizontal position (Switch 1, 3, 5) were almost identical. Since all parameters except PT are identical across switches, PT is the only factor that can change RTs when switches are pressed with the same RT and speed. Therefore, we did not expect a significant difference in the mean RTs between switches with approximately the same PT, unless participants' behaviors are modulated. Therefore, if a significant difference in RTs is detected depending on switch position, it is likely this modulated the participant's behavior. In that case, switch position may also have affected EEG results, therefore, this analysis was performed to investigate the possible confounder of position effects.

To evaluate the effect of switch types on participant behavior, the RTs of *Good* trials and the obtained points in the task were submitted to linear mixed effect models (LME). The model included switch types, sessions, and interactions as fixed effects. Participants were processed as a random effect. The model for RTs in the Wilkinson notation is *RT*~*switch*+*session*+*switch*:*session*+(1∣*participants*) (Model 1). The same model was fitted using points in the sessions as a target variable.

To identify the effects of switch type on EEG responses, we fitted an LME per EEG response. An effect of RTs was also included for investigating effects of participants' performance on EEG responses. Participants were processed as a random effect. The model in the Wilkinson notation is *EEG*~*switch*+*session*+*RT*+*RT*:*session*+*switch*:*session*+*switch*:*RT*+*switch*:*session*:*RT*+(1∣*participants*) (Model 2).

A questionnaire survey was administered to investigate whether it could detect differences in the psychological feeling of switch pressing. Switch rankings were submitted to LME analysis as a target variable per question item. Since participants' performances may have affected the detection of differences in psychological feelings across switch types, the model included switch types, RTs, and their interactions as fixed effects. Since the questionnaire survey was performed after all sessions, RTs were averaged across sessions for this analysis. Participants were processed as a random effect. The Wilkinson notation of the model is *ranking*~*switch*+*RT*+*switch*:*RT*+(1∣*participants*) (Model 3). To control for the false discovery rate across question items, *p*-values were corrected using the Benjamini-Hochberg method (Benjamini and Hochberg, 1995). In both analyses of EEG responses and questionnaires, RT data were centered using the mean value.

For LME modeling, the R software (R Core Team, 2020) and an *lme4* package (Bates et al., 2015) were used. The type II Wald χ^2^ tests were used to determine the significance of the fixed effects based on *car* packages (Fox and Weisberg, 2019). For the *post-hoc* tests, the multiple comparisons of switch types or sessions were performed based on the fitted LMEs with the Kenward-Roger method and the Tukey method to correct *p*-values for multiple comparisons, if necessary. For the significant interaction of RTs by sessions or switch types in LME analysis of EEG responses or questionnaire surveys, the significance of a coefficient of a RT trend per level of the factor and the differences in coefficients across levels were tested. All *post-hoc* tests were performed using the *emmeans* packages (Lenth, 2021).

## 3. Results

### 3.1. Behavioral Data

To investigate whether switch positions significantly affected participant performance, a paired *t*-test was performed based on the mean RTs between switch positions (upper/lower or horizontal position). The result showed that the RTs of the upper/ lower switches were almost significantly longer than horizontally-positioned switches (*t* = 1.97, *p* = 0.06, Cohen's *d* = 0.39), suggesting that switch position affected the participants' behavioral performance. As this effect is not intrinsically related to the mechanical parameters of the switches, we decided to use only switches arranged in the same direction. To use as many switches with equally spaced PTs as possible, and considering that switches 2 and 4 have relatively small PT deviations from Switch 3 with a normal PT, horizontally arranged switches (i.e., Switch 1, 3, and 5) were submitted to further analysis.

**Figure 3:**
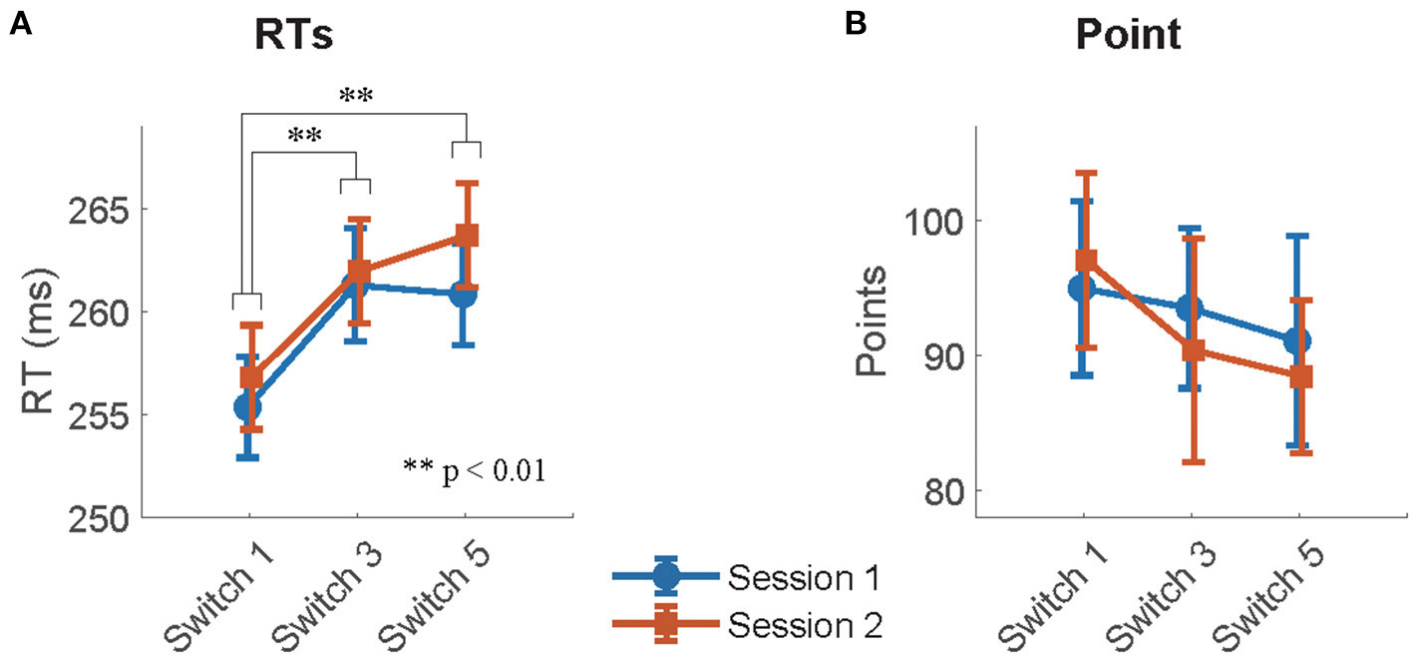
**(A)** The mean RTs of *Good* trials and **(B)** mean points per session and switch type.

The mean RTs of *Good* trials and mean points earned in each session across participants in the remaining switches are summarized in Figures 3A,B for both sessions, respectively. The effect of switch type on participants' behavioral performance was investigated using Model 1, which analyzed the mean RTs of *Good* trials and the mean points earned in sessions as a target variable, respectively, and switch types (switch: Switch 1, 3, and 5) and sessions (session: 1 and 2), and their interactions as fixed effects. For RTs, the type II Wald χ^2^ test revealed a significant effect of switch type [switch: χ^2^_(2)_ = 27.52, *p* < 0.01]. Multiple comparisons with *p*-value corrections based on the Tukey method revealed that the mean RTs of Switch 1 were significantly quicker than those of Switch 3 and 5 (Switch 1–Switch 3: *t* = −4.19, *p* < 0.01, Switch 1–Switch 5: *t* = −4.68, *p* < 0.01). On the other hand, no significant differences were observed between Switch 3 and 5 (Switch 3–Switch 5: *t* = −0.49, *p* = 0.88). The effect of sessions [session: χ^2^_(1)_ = 2.47, *p* = 0.12] and the interaction of switches by sessions [switch × session: χ^2^_(2)_ = 0.72, *p* = 0.70] did not reach significance. For task points, no fixed effect reached significance [switch: $\chi_{\left(2\right)}^{2}$ = 2.52, *p* = 0.28, session: $\chi_{\left(1\right)}^{2}$ = 0.14, *p* = 0.71, switch × session: $\chi_{\left(2\right)}^{2}$ = 0.51, *p* = 0.77]. LME analysis and Wald χ^2^ tests for RTs and points are summarized in Tables 2, 3.

**Table 2:** Estimated coefficients of fixed effects in LMEs for RTs and points.

	**RTs**		**Points**	
**Fixed effects**	**Estimates**	**CI**	**Estimates**	**CI**
Switch 1 (Intercept)	255.37	[250.50, 260.25]	95.01	[81.88, 108.14]
Switch 3	5.92	[2.33, 9.51]	−1.47	[−12.56, 9.62]
Switch 5	5.48	[1.89, 9.07]	−3.90	[−14.99, 7.19]
Session 2	1.45	[−2.14, 5.04]	2.07	[−9.01, 13.16]
Switch 3:Session 2	−0.77	[−5.85, 4.31]	−5.20	[−20.88, 10.48]
Switch 5:Session 2	1.40	[−3.68, 6.48]	−4.70	[−20.37, 10.98]

*CI denotes a 95% confidence interval*.

**Table 3:** Summaries of the Wald χ^2^ tests in the LMEs for RTs and points.

	**RTs**			**Points**		
**Fixed effects**	**χ^2^**	***df***	***p***	**χ^2^**	***df***	***p***
Switch	27.52	2	<0.01^**^	2.52	2	0.28
Session	2.47	1	0.12	0.14	1	0.71
Switch:Session	0.72	2	0.70	0.51	2	0.77

*df, degrees of freedom*.

** *p < 0.01*.

### 3.2. Pre-cue Onset

#### 3.2.1. P3

**Figure 4:**
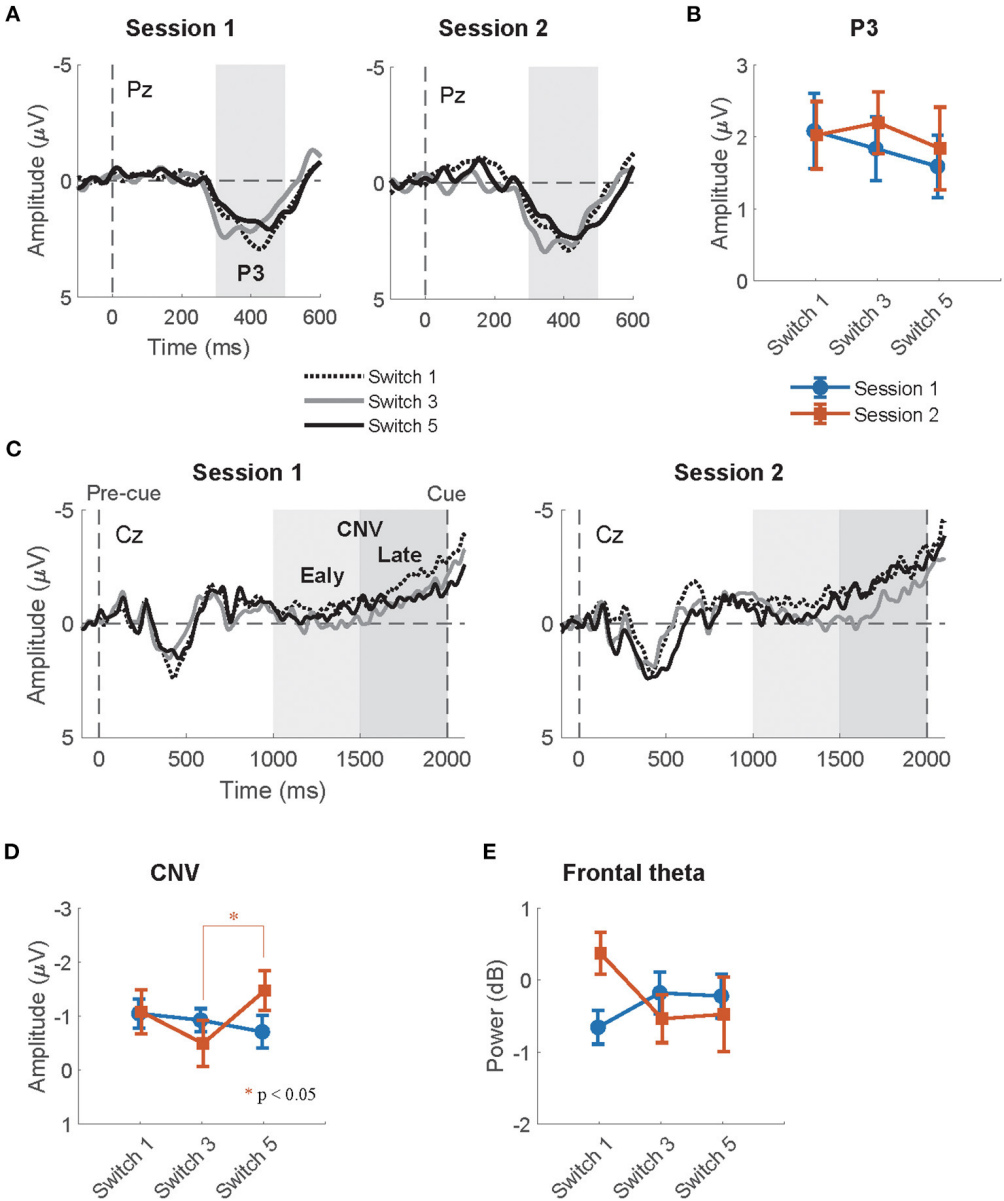
**(A)** The grand-average ERP relative to pre-cue onsets at Pz per session and switch type. The highlighted time window was used for the calculation of the mean amplitudes of P3. **(B)** The mean amplitude of the P3 relative to pre-cue onsets at Pz per session and switch type. **(C)** The grand-average CNV waveforms relative to pre-cue onset at Cz per session and switch type. The time windows of the early and late CNV are highlighted in light and dark gray, respectively. **(D)** The mean amplitudes of the normalized CNV at Cz per session and switch type. **(E)** The mean frontal theta power (dB) at Fz per session and switch type.

The summaries of LME fittings and Wald χ^2^ tests for all EEG responses relative to pre-cue onsets are summarized in Tables 4, 5, respectively. Figure 4A shows the grand-average ERP at Pz relative to the pre-cue onset per session and switch type. The effects of switch type, participants' performance (RTs), and sessions on mean P3 amplitudes were investigated using Model 2. The Wald χ^2^ test showed that an almost significant interaction of switch types by RTs [switch × RT: χ^2^_(2)_ = 5.12, *p* = 0.08]. The *post-hoc* tests revealed that the coefficient of an RT trend did not reach significance for any of the switches (Switch 1: coeff. = −0.02 ± 0.02, *t* = −0.72, *p* = 0.47; Switch 3: coeff. = 0.03 ± 0.02, *t* = 1.54, *p* = 0.13, Switch 5: coeff. = 0.01 ± 0.02, *t* = 0.67, *p* = 0.50). In addition, there were no significant differences in the coefficients of RT trends across the switches (Switch 1–Switch 3: *t* = −2.14, *p* = 0.09, Switch 1–Switch 5: *t* = −1.34, *p* = 0.38, Switch 3–Switch 5: *t* = 0.77, *p* = 0.72). Other fixed effects did not reach significance in LME analysis (see Table 5 for details). The mean P3 amplitudes by switch and session are depicted in Figure 4B.

**Table 4:** Estimated coefficients of fixed effects in LMEs for EEG responses relative to pre-cue.

	**P3**		**CNV**		**Frontal theta**	
**Fixed effects**	**Estimates**	**CI**	**Estimates**	**CI**	**Estimates**	**CI**
Switch 1 (Intercept)	2.07	[1.14, 3.01]	−0.86	[−1.51, −0.21]	−0.60	[−1.27, 0.08]
Switch 3	−0.28	[−1.06, 0.51]	−0.06	[−0.88, 0.75]	0.40	[−0.50, 1.30]
Switch 5	−0.49	[−1.27, 0.29]	0.11	[−0.71, 0.92]	0.38	[−0.52, 1.27]
Session 2	−0.16	[−0.94, 0.62]	−0.17	[−0.99, 0.66]	0.98	[0.07, 1.89]
RT	0.00	[−0.05, 0.05]	0.04	[−0.01, 0.09]	0.01	[−0.04, 0.07]
Session 2:RT	−0.03	[−0.09, 0.03]	−0.03	[−0.09, 0.03]	−0.01	[−0.08, 0.06]
Switch 3:Session 2	0.50	[−0.59, 1.58]	0.49	[−0.65, 1.64]	−1.24	[−2.50, 0.02]
Switch 5:Session 2	0.32	[−0.77, 1.42]	−0.71	[−1.86, 0.44]	−1.05	[−2.32, 0.23]
Switch 3:RT	0.03	[−0.03, 0.09]	−0.04	[−0.10, 0.02]	0.00	[−0.07, 0.07]
Switch 5:RT	0.01	[−0.05, 0.07]	0.00	[−0.06, 0.07]	−0.02	[−0.09, 0.05]
Switch 3:Session 2:RT	0.03	[−0.05, 0.11]	0.09	[0.00, 0.18]	−0.05	[−0.15, 0.04]
Switch 5:Session 2:RT	0.05	[−0.04, 0.13]	0.02	[−0.07, 0.11]	−0.04	[−0.14, 0.06]

*CI denotes a 95% confidence interval*.

**Table 5:** Summaries of the Wald χ^2^ tests in the LMEs for EEG responses relative to pre-cue.

	**P3**			**CNV**			**Frontal theta**		
**Fixed effects**	**χ^2^**	***df***	***p***	**χ^2^**	***df***	***p***	**χ^2^**	***df***	***p***
Switch	2.37	2	0.31	2.15	2	0.34	0.24	2	0.89
Session	0.57	1	0.45	0.55	1	0.46	0.42	1	0.52
RT	0.61	1	0.44	7.24	1	<0.01^**^	0.94	1	0.33
Session:RT	0.03	1	0.86	0.26	1	0.61	3.83	1	0.05^†^
Switch:Session	0.56	2	0.76	5.55	2	0.06^†^	3.65	2	0.16
Switch:RT	5.12	2	0.08^†^	0.64	2	0.73	2.30	2	0.32
Switch:Session:RT	1.19	2	0.55	4.19	2	0.12	1.23	2	0.54

*df, degrees of freedom*.

† *p < 0.10*,

** *p < 0.01*.

#### 3.2.2. CNV

The grand-average CNV at Cz per session and switch type is shown in Figure 4C. The results of the Wald χ^2^ test showed that a significant effect of RTs [RT: χ^2^_(1)_ = 7.24, *p* < 0.01]. All coefficients of RT trends showed a positive relationship between CNV amplitudes and RTs (c.f. Table 4). Since the enhancement in mean CNV amplitudes shows more negative deflection, this positive correlation indicates that quicker RTs increase mean CNV amplitudes. An almost significant interaction of switch types by sessions [Switch × session: χ^2^_(2)_ = 5.55, *p* = 0.06] was found. Multiple comparisons of mean CNV amplitudes across switch types per session revelated that larger mean amplitudes of Switch 5 (Longest PT) than those of Switch 3 (Normal PT) in session 2 (Switch 3–Switch 5: *t* = 2.39, *p* < 0.05). There were no other significant differences in session 1 (Switch 1–Switch 3: *t* = 0.15, *p* = 0.99, Switch 1–Switch 5: *t* = −0.25, *p* = 0.97, Switch 3–Switch 5: *t* = −0.41, *p* = 0.91) and session 2 (Switch 1–Switch 3: *t* = −1.00, *p* = 0.58, Switch 1–Switch 5: *t* = 1.36, *p* = 0.36). The *p*-values were corrected using the Tukey method. Other fixed effects did not reach significance in LME analysis (see Table 5 for the details). The mean CNV amplitudes per switch and session are depicted in Figure 4D.

#### 3.2.3. Frontal Theta

The mean frontal theta power at Fz per session and switch type is depicted in Figure 4E. The Wald χ^2^ tests showed that the interaction of sessions by RTs was almost significant [session × RT: χ^2^_(1)_ = 3.83, *p* = 0.05]. The *post-hoc* tests showed that a coefficient of a RT trend in session 2 was similarly almost significant, but did not reach significance (session 1: coeff. = 0.01 ± 0.02, *t* = 0.50, *p* = 0.62, session 2: coeff. = −0.03 ± 0.02, *t* = −1.90, *p* = 0.06). The difference in the coefficient of RT trends between session 1 and 2 was almost significant (session 1–session 2: *t* = 1.82, *p* = 0.07). Any other fixed effects also showed no significant effects in LME analysis (see Table 5 for details).

### 3.3. Feedback Onset

#### 3.3.1. N1

**Figure 5:**
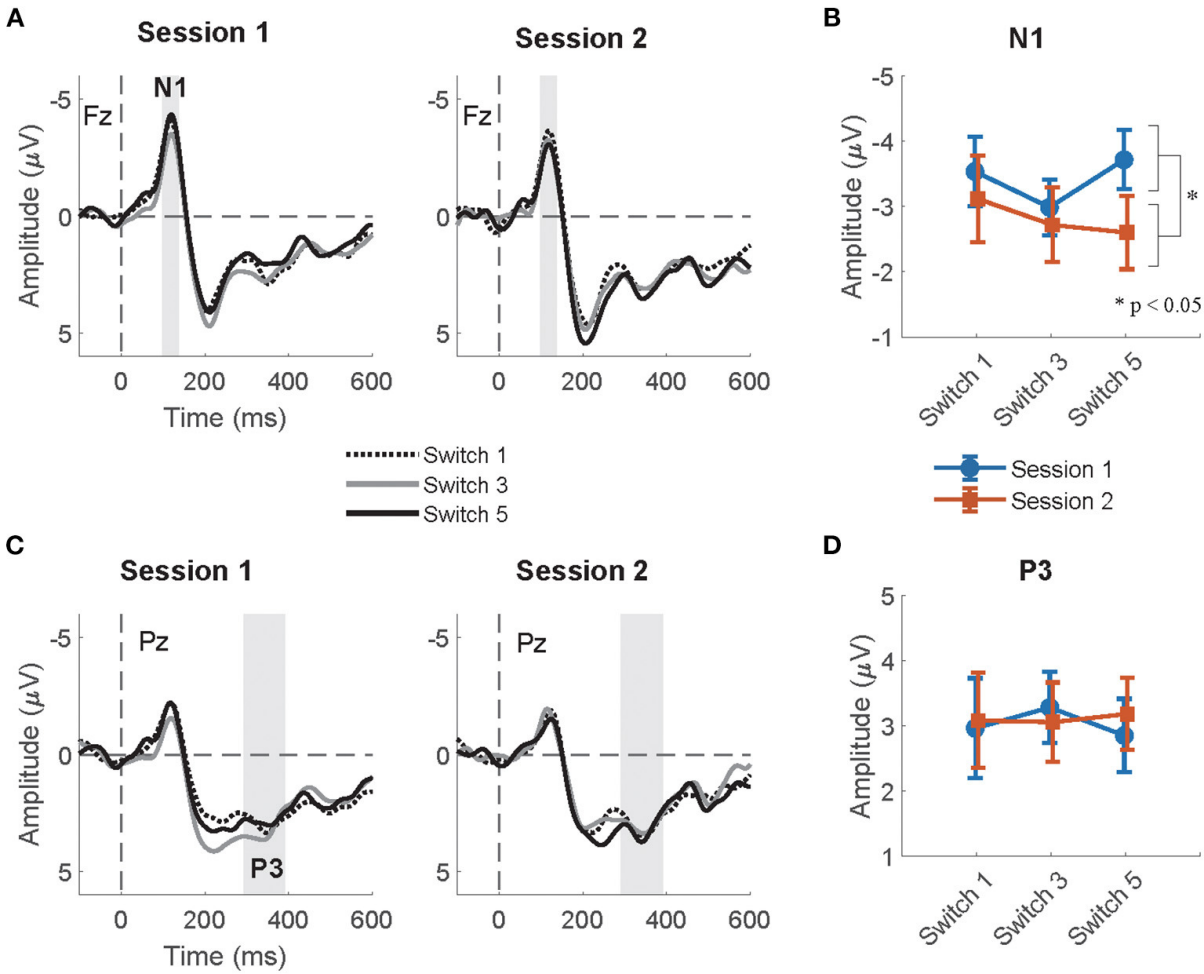
**(A)** The grand-average ERP relative to feedback onset at Fz per session and switch type. The highlighted time window was used for the calculation of the mean amplitudes of N1. **(B)** The mean amplitude of the N1 relative to feedback onsets at Fz per session and switch type. **(C)** The grand-average ERP relative to feedback onset at Pz per session and switch type. The highlighted time window was used for the calculation of the mean amplitudes of P3. **(D)** The mean amplitude of the P3 relative to feedback onsets at Pz per session and switch type.

The summaries of LME fittings and Wald χ^2^ tests for all EEG responses relative to feedback onsets are summarized in Tables 6, 7, respectively. The grand-average ERP at Fz relative to feedback onsets per session and switch type is shown in Figure 5A. The Wald χ^2^ tests showed a significant effect of sessions [session: χ^2^_(1)_ = 5.84, *p* = 0.02]. The *post-hoc* test showed significantly larger mean amplitudes in session 1 than in session 2 (session 1–session 2: *t* = −2.17, *p* = 0.03). Any other fixed effects showed no significant effects in LME analysis (see Table 7 for details). The mean amplitudes per switch and session are depicted in Figure 5B.

**Table 6:** Estimated coefficients of fixed effects in LMEs for EEG responses relative to feedback onset.

	**N1**		**P3**	
**Fixed effects**	**Estimates**	**CI**	**Estimates**	**CI**
Switch 1 (Intercept)	−3.54	[−4.61,−2.46]	2.91	[1.65, 4.17]
Switch 3	0.58	[−0.31, 1.46]	0.37	[−0.78, 1.51]
Switch 5	−0.18	[−1.06, 0.70]	−0.05	[−1.20, 1.09]
Session 2	0.40	[−0.47, 1.28]	0.18	[−0.96, 1.32]
RT	0.00	[−0.06, 0.06]	−0.01	[−0.09, 0.07]
Session 2:RT	−0.01	[−0.08, 0.06]	0.02	[−0.08, 0.11]
Switch 3:Session 2	−0.17	[−1.38, 1.04]	−0.35	[−1.93, 1.22]
Switch 5:Session 2	0.68	[−0.54, 1.90]	0.28	[−1.31, 1.87]
Switch 3:RT	−0.02	[−0.09, 0.05]	0.01	[−0.08, 0.09]
Switch 5:RT	0.00	[−0.07, 0.07]	0.00	[−0.09, 0.09]
Switch 3:Session 2:RT	0.03	[−0.06, 0.13]	−0.04	[−0.17, 0.08]
Switch 5:Session 2:RT	0.01	[−0.08, 0.11]	−0.04	[−0.17, 0.09]

*CI denotes a 95% confidence interval*.

**Table 7:** Summaries of the Wald χ^2^ tests in the LMEs for EEG responses relative to feedback onset.

	**N1**			**P3**		
**Fixed effects**	**χ^2^**	***df***	***p***	**χ^2^**	***df***	***p***
Switch	2.46	2	0.29	0.29	2	0.86
Session	5.84	1	0.02^*^	0.10	1	0.75
RT	0.02	1	0.89	0.37	1	0.54
Session:RT	0.22	1	0.64	0.23	1	0.63
Switch:Session	1.99	2	0.37	0.65	2	0.72
Switch:RT	0.30	2	0.86	0.53	2	0.77
Switch:Session:RT	0.41	2	0.81	0.57	2	0.75

*df, degrees of freedom*.

* *p < 0.05*.

#### 3.3.2. P3

Figure 5C shows the grand-average ERP at Pz relative to the feedback onset per session and switch type. The Wald χ^2^ test showed no significant results in any fixed effects (see Table 7 for the details). The mean P3 amplitudes per switch and session are depicted in Figure 5D.

### 3.4. Questionnaire

**Figure 6:**
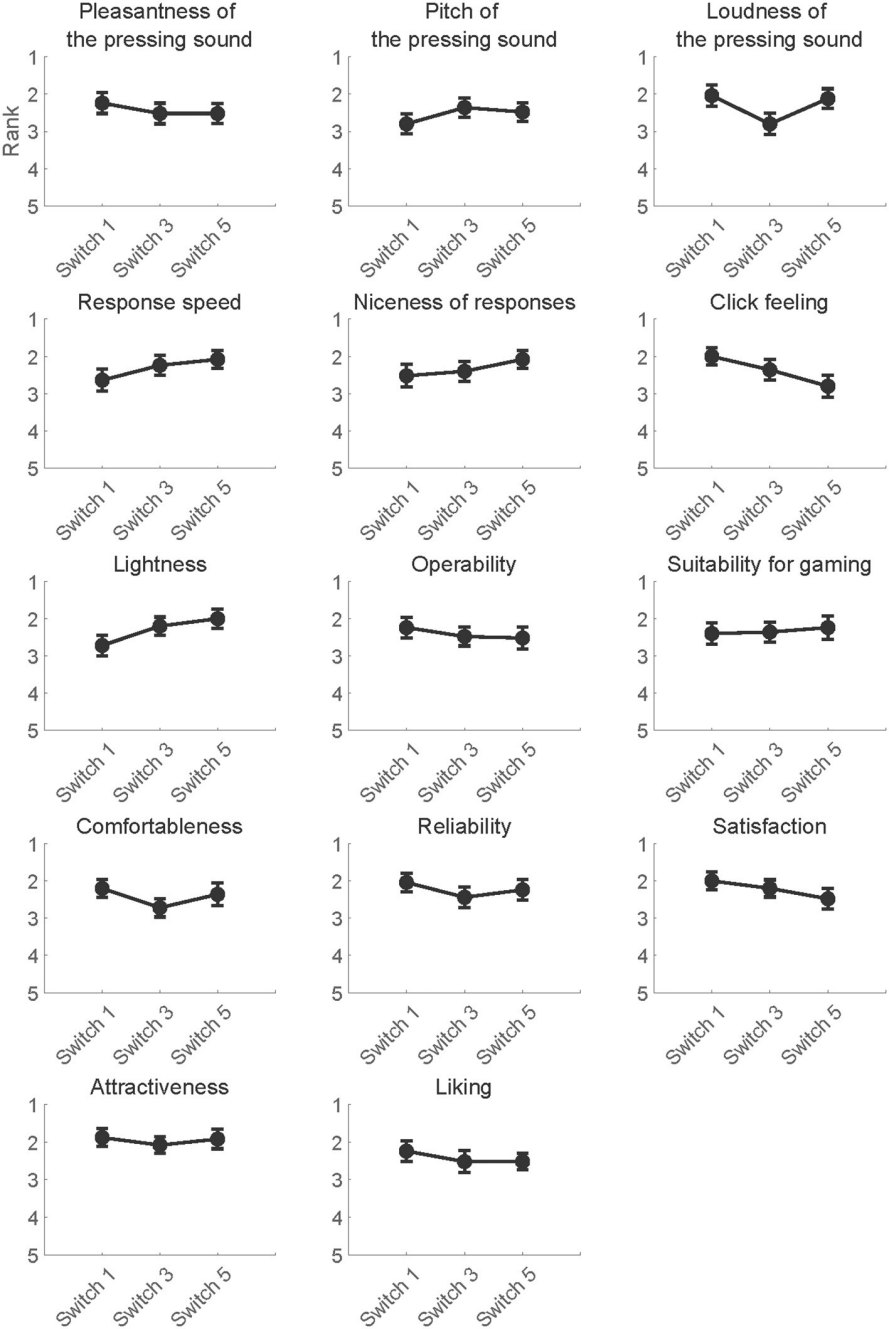
The mean subjective rankings of switches per question item.

The mean rankings per question item are summarized in Figure 6. To assess whether participants consciously perceived differences in the psychological perception of switches, the ranking data in the questionnaire survey were used to construct Model 3 per question item. The models included questionnaire rankings as a target variable, switch types, RTs and its interaction as fixed effects. The *p*-values of fixed effects were adjusted using the false discovery rate across question items according to the BH-method (Benjamini and Hochberg, 1995). Any fixed effects in every question item did not reach significance (*p* > 0.05). The results of the Wald χ^2^ tests are summarized in Table 8.

**Table 8:** Summaries of the Wald χ^2^ tests in the LMEs for question item in the subjective evaluation.

	**Switch**			**RT**			**Switch** × **RT**		
**Question item**	**χ^2^**	***df***	***p***	**χ^2^**	***df***	***p***	**χ^2^**	***df***	***p***
Pleasantness of the pressing sound	1.30	2	0.79	1.21	1	0.64	4.40	1	0.37
Pitch of the pressing sound	1.07	2	0.79	1.13	1	0.64	2.83	1	0.48
Loudness of the pressing sound	6.42	2	0.32	1.29	1	0.64	0.99	1	0.85
Response speed	2.90	2	0.66	0.00	1	0.99	0.47	1	0.96
Niceness of responses	1.17	2	0.79	0.61	1	0.64	3.92	1	0.37
Click feeling	6.19	2	0.32	0.86	1	0.64	0.23	1	0.96
Lightness	3.98	2	0.64	0.96	1	0.64	1.91	1	0.67
Operability	0.45	2	0.86	0.33	1	0.72	6.39	1	0.29
Suitability for gaming	0.08	2	0.96	1.22	1	0.64	1.49	1	0.74
Comfortableness	3.02	2	0.66	0.77	1	0.64	6.43	1	0.29
Reliability	0.95	2	0.79	0.56	1	0.64	3.67	1	0.37
Satisfaction	1.85	2	0.79	0.03	1	0.93	0.04	1	0.98
Attractiveness	0.74	2	0.81	0.11	1	0.86	0.29	1	0.96
Liking	1.71	2	0.79	0.70	1	0.64	4.14	1	0.37

*df, degrees of freedom. p-values were adjusted by the BH-method*.

## 4. Discussion

The current research aimed to elucidate whether differences in the PTs of switches modulate the late CNV amplitudes associated with motor preparation to press mechanical switches. This research was underpinned by the motivation to incorporate the allocated attentional resources to switch operation into the evaluation of mechanical switch parameters for gaming keyboards. We predicted that switches with PT values deviating from the normal range would increase the attentional resources allocated to switch operation, which would be reflected in a mean CNV amplitude increase (i.e., mean CNV amplitude of Switch 1, Switch 5 > Switch 3).

In line with our prediction, we observed that the mean amplitude of the normalized CNV in Switch 5 (longest PT) was significantly larger than that in Switch 3 (medium PT among the experimental switches studied herein) in the second half session. Given that attention increases correlate with the enhancement of late CNV amplitudes (McCallum and Walter, 1968; Tecce, 1972; Liebrand et al., 2017), the longest PT of Switch 5, which considerably deviates from that of commonly used switches, may have increased attention to the switch during motor preparation. The switch type effects on mean CNV amplitudes were observed only in the second half sessions. Participants might have gradually noticed the differences and modulated their attention to switch operation as the sessions progressed. The results that the mean N1amplitude relative to auditory feedback, which is enhanced by top-down attention to auditory input (Hillyard et al., 1973; Näätänen and Picton, 1987), was significantly larger in session 1 than in session 2 might also suggest that the gap increased their attentions to the feedback at the beginning of the task. In addition, while other EEG responses (N1, P3, and frontal theta) to pre-cue and feedback onsets were also measured in the current research, these responses did not significantly differ between switches in *post-hoc* tests. Thus, among all EEG responses measured in the current research, CNV seems to be the most reliable feature for detecting increases in attention allocation to switch operations.

We predicted a significant difference between Switch 1 and 3, but the difference did not reach statistical significance. However, the mean CNV amplitude of Switch 1 tended to be larger than that of Switch 3. Since the RT of Switch 1 was significantly quicker than that of the other switches due to the short PT and RTs are correlated with sustained attention in reaction tasks (Buck, 1966), the attention increase due to PT deviation may have been smaller than that of Switch 5 because a faster RT with Switch 1 can be more easily obtained than with Switch 5. In addition, the small number of participants in the current study, which resulted in a medium detectable effect size, may have contributed to the lack of a significant difference. Since we cannot confirm a difference between Switch 1 (shortest PT) and Switch 3 (normal PT) in the current study, an experiment with more participants is warranted.

The results of the questionnaire survey showed no significant effect of the type of switch on the subjective switch ranking. This suggests that PT differences and the corresponding increase in attentional resources cannot be extracted by questionnaire surveys, which supports the usefulness of brain activity characterization for marketing purposes. In particular, among the measures of brain activity used in neuromarketing, the use of EEG has an advantage in terms of evaluation in real-world environments. Indeed, EEG is preferred in various marketing conditions because its measurement systems have developed in recent years and permit wearable measuring devices that are compact and do not require application of conductive gels on the user's scalp (Higashi et al., 2017). To date, EEG measurements have been widely demonstrated to be a powerful tool with high temporal resolution for neuromarketing studies (Dmochowski et al., 2014; Golnar-Nik et al., 2019; Aldayel et al., 2020). As an extension of these EEG-based neuromarketing studies, the current research fulfilled the purpose of demonstrating that EEG measurements have the potential to provide a human-centric evaluation of the PTs of mechanical switches for gaming keyboards in a way that does not impose extra allocation of attentional resources to switch operation.

Since Switch 1 showed the shortest RT and it does not significantly increase the attentional resources compared to a switch with normal PT value (Switch 3), in terms of behavioral data and attentional resources, the switch with the shortest PT seems to be the best one in the current study. However, in game situations that require more complex switch operations than the current reaction task, switches with shorter PTs are more prone to operation errors because when a finger is placed on a switch, subtle finger movements unrelated to switch pressing are often misinterpreted as input. In addition, such characteristics may also cause fatigue when gaming for long periods of time since users continue to pay attention to the switch so as not to produce errors. In the current study, we demonstrated that PT length induces an increase in the attentional resources allocated to switch pressing, and that there is no significant increase for a switch with short PT. On the other hand, the pursuit of RT alone does not necessarily lead to the development of optimal switches. Therefore, a multifaceted evaluation of mechanical switches, including the attentional resources allocated to switch operation in addition to RT, will lead to more human-centered product development. In the future, it may be possible to evaluate a newly developed switch in terms of the attentional resources allocated to its operation. Alternatively, when selecting which mechanical switch to use in a computer game, selecting that with the lowest CNV among several with different parameters may prevent the increase in the attention assigned to switch operations.

The current study has several limitations. First, during recruitment, the absence of motor disorders was not considered as selection criterion. Thus, if participants with a history of such disorders were involved in the current study, motor preparation might have been affected. Second, due to the low number of participants in the current study, only a medium effect size could be detected in case of a paired *t*-test. Thus, the lack of significant difference of mean CNV amplitudes between Switch 1 and 3 was probably due to the small number of subjects. Thus, in the future it will be necessary to investigate the switch effects on mean CNV amplitudes using a larger number of subjects.

## 5. Conclusions

In conclusion, the current research provides preliminary evidence that EEG is an effective evaluation criterion for mechanical parameters of switches in terms of the attentional resources allocated to switch operations. The results suggest our method to evaluate mechanical parameters can help prevent increasing attention allocation to switch operation. The wireless wearable EEG measurement system with few electrodes which we employed may be useful for parameter design in real-world environments. Further investigations using a larger sample size and data collection from more switch types are necessary to confirm the applicability of the current method to the actual design of mechanical switches for gaming.

## Data Availability Statement

The datasets presented in this article are not readily available because, the part of data supporting the conclusions of this article will be made available by request to the corresponding author. Requests to access the datasets should be directed to Yasushi Naruse, y_naruse@nict.go.jp.

## Ethics Statement

The studies involving human participants were reviewed and approved by the Ethical Committee for Human and Animal Research of the National Institute of Information and Communications Technology. The patients/participants provided their written informed consent to participate in this study.

## Author Contributions

KF and KNakat developed the experimental switches. HW, KNakaj, ST, RM, and MS performed the data collection. HW and KNakaj analyzed the data. HW, KNakaj, ST, RM, MS, YY, HK, HN, and YN discussed the results. HW wrote the manuscript. All authors discussed the experimental design and data collection procedures, reviewed and approved the manuscript.

## Conflict of Interest

The authors declare that this study received funding from OMRON Corporation. The funder had the following involvement with the study: the study design, preparation of the experimental devices, data collection, interpretation of data, and the decision to submit it for publication.

## Publisher's Note

All claims expressed in this article are solely those of the authors and do not necessarily represent those of their affiliated organizations, or those of the publisher, the editors and the reviewers. Any product that may be evaluated in this article, or claim that may be made by its manufacturer, is not guaranteed or endorsed by the publisher.

## Supplementary Material

Supplementary Figure 1 Representative parameters of the mechanical switches related to force required to press the switches and distances to move the switches from free positions (Stroke).
